# Chemically-Mediated Roostmate Recognition and Roost Selection by Brazilian Free-Tailed Bats (*Tadarida brasiliensis*)

**DOI:** 10.1371/journal.pone.0007781

**Published:** 2009-11-10

**Authors:** Amy C. Englert, Michael J. Greene

**Affiliations:** Department of Integrative Biology, University of Colorado Denver, Denver, Colorado, United States of America; Institute of Evolutionary Biology (CSIC-UPF), Spain

## Abstract

**Background:**

The Brazilian free-tailed bat (*Tadarida brasiliensis*) is an exceptionally social and gregarious species of chiropteran known to roost in assemblages that can number in the millions. Chemical recognition of roostmates within these assemblages has not been extensively studied despite the fact that an ability to chemically recognize individuals could play an important role in forming and stabilizing complex suites of social interactions.

**Methodology/Principal Findings:**

Individual bats were given a choice between three roosting pouches: one permeated with the scent of a group of roostmates, one permeated with the scent of non-roostmates, and a clean control. Subjects rejected non-roostmate pouches with greater frequency than roostmate pouches or blank control pouches. Also, bats chose to roost in the roostmate scented pouches more often than the non-roostmate or control pouches.

**Conclusions/Significance:**

We demonstrated that *T. brasiliensis* has the ability to chemically recognize roostmates from non-roostmates and a preference for roosting in areas occupied by roostmates. It is important to investigate these behaviors because of their potential importance in colony dynamics and roost choice.

## Introduction

Bats can distinguish between roostmates and non-roostmates, males and females, and individuals of different age based on scent alone [1], [2], [3]. The recognition of roostmates allows philopatric colonial bat species to form stable associations within closed colonies or harems [1], [4], [5], [6], [7]. Bats that live in a group often exhibit a group scent profile in addition to an individual scent profile which can be used for identification by other individuals [8]. Scent profiles can be created by a combination of scents produced by glandular secretions, urine, feces, and the microbial action of bacterial communities [9].

There is evidence that chemical communication is used extensively in bats to mediate social interactions, but little is known about how chemical signals might affect roosting choices [7], [8]. If wild bats can distinguish between familiar and unfamiliar individuals based on odor cues, this could contribute to philopatry and colony stability.

The purpose of this study was to show that Brazilian free-tailed bats (*Tadarida brasiliensis*) distinguish between roost-mates and non-roost-mates using chemical cues alone. The test species is a medium-sized microchiropteran that is well known for maternity colonies exceeding millions of individuals in size [10]. The species has a unique and pungent scent profile and females have demonstrated the ability to recognize individuals chemically [10], [11].

## Results

**Figure 1:**
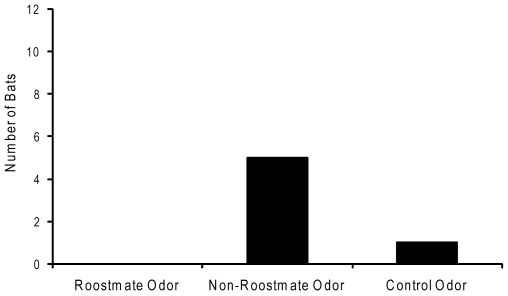
Pouch rejection by *Tadarida brasiliensis*. During the experiment, 6 of 12 bats exhibited pouch rejection behavior prior to roosting inside, under, or behind another pouch. This figure shows the number of bats that rejected pouches treated with roostmate odors, non-roostmate odors, or a clean control pouch. More bats rejected pouches containing non-roostmate odors than pouches containing roostmate odors or blank control pouches (X^2^ = 7.001, *p* = 0.0151).

Six of 12 bats exhibited pouch rejection behavior. Of those bats, more rejected pouches containing non-roostmate odors than pouches containing roostmate odors or blank control pouches (X^2^ = 7.001, *p* = 0.0151; Fig. 1). Bats rejected pouches containing non-roostmate odors more than pouches containing roostmate odors (X^2^ = 5.000, *p* = 0.0127, Bonferroni corrected α = 0.017). There was no significant difference in the rejection rates between pouches containing roostmate odors and blank control pouches (X^2^ = 1.000, *p* = 0.15, Bonferroni corrected α = 0.017) or between non-roostmate pouches and control pouches (X^2^ = 2.667, *p* = 0.051, Bonferroni corrected α = 0.017).

**Figure 2:**
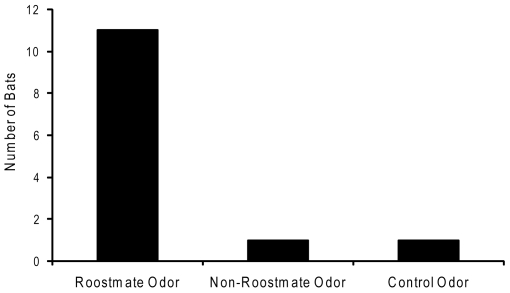
Chemically-mediated roosting choices by *Tadarida brasiliensis*. The figure shows the number of bats that entered or burrowed under or behind pouches treated with roostmate odors, non-roostmate odors, or a clean control pouch. More bats went in, under, or behind pouches with roostmate odors compared to either pouches containing non-roostmate odors (X^2^ = 8.333, *p* = 0.00195, Bonferroni corrected α = 0.017) or the control pouch (X^2^ = 8.333, *p* = 0.00195, Bonferroni corrected α = 0.017).

There was a significant difference in the number of bats which went in, under, or behind the pouches among the experimental treatments (X^2^ = 15.396, *p* = 0.00025; Fig. 2). More bats went in, under, or behind pouches with roostmate odors compared to either pouches containing non-roostmate odors (X^2^ = 8.333, *p* = 0.00195, Bonferroni corrected α = 0.017) or the control pouch (X^2^ = 8.333, *p* = 0.00195, Bonferroni corrected α = 0.017). There was no significant discrimination between the non-roostmate odor treated and control pouches (X^2^ = 0, *p* = 0.5, Bonferroni corrected α = 0.017).

## Discussion

Our data show that *T. brasiliensis* bats can discriminate between roostmates and non-roostmates using chemical odors alone. Although this is a highly gregarious and colonial species, most of the focal animals refused to roost in a pouch which contained non-roostmate odors when given a choice including a control pouch and one containing a roostmate odor.

Our data demonstrate that the bats also make roosting choices based on their perception of chemical roostmate recognition cues. Bats entered and burrowed under or behind pouches containing roostmate odor more often than either pouches with non-roostmate odor or control pouches throughout the course of the experiment. It is not surprising that test bats preferred to roost in pouches which contain the odors of familiar individuals, but it is remarkable that they showed the ability to detect differences in colony scent profiles, despite the fact that all the bats in both colonies subsist on the same diet, have similar roosting conditions, and bats within colonies are not related. More research will be necessary to understand the mechanism, such as phenotype matching, by which the bats make recognition decisions.

Our results contribute to a rather limited data set regarding chemical recognition of roostmates in bats. De Fanis and Jones [3] demonstrated the ability of common pipistrelles (*Pipistrellus pipistrellis*) to discern between roostmate and non-roostmate odors, but they used a positive reinforcement training method rather than the observation of normal behaviors, and tested the bats using the binomial choice of a y-maze. Additionally, these bats were taken from the same natural colonies, so their genetic relationship is not known. Bouchard [2] studied sex and roostmate recognition in Angolan free-tailed bats (*Mops condylurus*) and little free-tailed bats (*Chaerephon pumilus*), but also designed a study with an arena which tested a binomial choice using roostmates from a natural population. Bloss et al. [1] determined that big brown bats (*Eptesicus fuscus*) had the ability to distinguish between familiar and unfamiliar odors, but again their study was conducted in a y-maze.

Our data are novel because they demonstrate not only the capability of *T. brasiliensis* to discern between roostmate and non-roostmate odors, but also that it is relevant to them behaviorally, as they will exhibit the behavior in a free-choice environment (where they can also choose a control pouch or no pouch). The study of chemical communication is vital to the understanding of bat behaviors ranging from foraging and roosting to mating and pup-rearing. There have been only a few studies of this behavior to date [8], [10], [11]. Populations of bats cannot be managed effectively without an understanding of colony dynamics and roost choice, topics that can begin to be addressed through study of chemical communication among conspecifics.

## Materials and Methods

Experiments were done using two colonies of captive *T. brasiliensis*. Colony A was maintained at Bat World Sanctuary (Mineral Wells, Texas, U.S.A.) and colony B was maintained by Barbara French (Austin, Texas, U.S.A.). Animals used in the study were captive bats that were were mobile and exhibited normal behaviors. Bats of both colonies were from the same geographical area in Texas, subsisted on similar diets, and lived in similar captive conditions. All focal animals were adults capable of reproduction, although the study was carried out during the non-breeding season for this species.

Colony A was housed in an open roosting cage (approximately 1×1.5×0.5 m) within a larger enclosed indoor flight cage (approximately 3×3×8 m) which contained about fifty *T. brasiliensis*. Roosting pouches were attached to the walls and ceiling of the cage, and all bats were free to move throughout the cage. Temperature was held at approximately 22°C by air conditioning and heating, light intensity was kept dim during the day and dark at night (with a photoperiod of approximately 15 hours), and bats were fed mealworms *ad libitum* and, if needed, supplemented with a blended food mixture up to twice daily.

Colony B was housed in a small climate-controlled barn. Males and females were segregated in separate cages with roosting pouches attached to walls and ceilings. The barn was cooler than outside ambient temperature during the day, and roughly equaled ambient night temperatures (approximately 23°C) when the experiments were conducted. Light conditions inside the barn were kept uniformly dim through the day and night by covering the windows and providing dim artificial lighting. Bats were fed as described for colony A, although each bat was also hand-fed nightly.

Roosting pouches were constructed of thickly padded quilted cotton to produce a small pouch that is open on one end, much like a thumbless oven mitten, with dimensions of 19×23×5 cm. The bats roost inside, underneath, or behind roosting pouches.

Pieces of black felt, with dimensions of 12×17 cm, were used to absorb scents from each colony. Using felt to absorb scents was considered appropriate because during some parts of the year (e.g. hibernacula in winter), natural colonies contain animals of all ages and sexes, all of which would contribute to an overall colony scent profile. Fifty felt pieces were placed in roosting pouches of colony A for 5 days to create roostmate odor stimuli. On the same day, fifty pieces of felt were placed in male and female roosting pouches of colony B for 5 days to create non-roostmate odor stimuli. Felt was stored in sealed plastic bags in a refrigerator. Clean, blank control felt pieces were placed in a separate sealed bag and stored.

All experimental trials were completed using twenty bats from colony A as focal animals. A small cage (approximately 70×40×40 cm) at the location of colony A was used as the testing arena. The arena, placed within a larger flight cage, was constructed of black mesh stretched around a PVC frame. Two pieces of felt were placed into roosting pouches prior to each trial. One pouch contained two pieces of felt treated with roostmate odor. A second pouch contained two pieces of felt treated with non-roostmate odors. A third pouch contained two pieces of clean, blank control felt. The three pouches were randomly placed in three of the four corners of the arena prior to introduction of the focal bat. To start a trial, a bat was randomly chosen from a transport container and released in the middle of the cage, facing away from the human observer. After each trial, the felt pieces were discarded and replaced and new felt pieces were used. Although the same pouches were used in all experimental trials, the pouches were replaced if urine or guano was present after the trial. The type of stimulus placed in each pouch was randomly chosen so that each pouch contained all stimuli types over the course of the experiment.

Data were collected during a thirty-minute period by observing the behavior of bats using incidental light and the muted light of a small flashlight. The number of bats that entered each pouch or burrowed under or behind each pouch along with the number of bats that rejected each pouch was measured. Bats were considered to have rejected a pouch if they approached the pouch entrance, stopped, and investigated the entrance before subsequently refusing to enter the pouch. During trials in which bats entered more than one pouch, the final pouch chosen was used in the data analysis because in all trials bats spent the most time in the last pouch chosen.

Trials in which bats were obviously alarmed by handling, such as when bats moved directly to one location in the testing arena after being released and stayed at that location for the entirety of the trial, were excluded from analysis. Twenty trials using different bats were run for the experiment. Data from seven bats were excluded because the bats were clearly alarmed.

All research protocols used in the study were approved by the University of Colorado Denver Institutional Animal Care and Use Committee under assurance #A3658-01. The research methods were designed to minimize handling of bats and adhere to handling protocols established in the Animal Behavior Society's *Guidelines for the Treatment of Animals in Behavioral Research and Teaching*.
